# Redox chemistry of molybdenum in natural waters and its involvement in biological evolution

**DOI:** 10.3389/fmicb.2012.00427

**Published:** 2012-12-21

**Authors:** Deli Wang

**Affiliations:** State Key Laboratory of Marine Environmental Science, Xiamen UniversityXiamen, China

**Keywords:** molybdenum, redox speciation, enzymes, ancient ocean, biological evolution

## Abstract

The transition element molybdenum (Mo) possesses diverse valances (+II to +VI), and is involved in forming cofactors in more than 60 enzymes in biology. Redox switching of the element in these enzymes catalyzes a series of metabolic reactions in both prokaryotes and eukaryotes, and the element therefore plays a fundamental role in the global carbon, nitrogen, and sulfur cycling. In the present oxygenated waters, oxidized Mo(VI) predominates thermodynamically, whilst reduced Mo species are mainly confined within specific niches including cytoplasm. Only recently has the reduced Mo(V) been separated from Mo(VI) in sulfidic mats and even in some reducing waters. Given the presence of reduced Mo(V) in contemporary anaerobic habitats, it seems that reduced Mo species were present in the ancient reducing ocean (probably under both ferruginous and sulfidic conditions), prompting the involvement of Mo in enzymes including nitrogenase and nitrate reductase. During the global transition to oxic conditions, reduced Mo species were constrained to specific anaerobic habitats, and efficient uptake systems of oxidized Mo(VI) became a selective advantage for current prokaryotic and eukaryotic cells. Some prokaryotes are still able to directly utilize reduced Mo if any exists in ambient environments. In total, this mini-review describes the redox chemistry and biogeochemistry of Mo over the Earth’s history.

## INTRODUCTION

Only a few transition elements (e.g., Fe, Mo, and Cu) were selected in the evolution of life and play a fundamental role in the global cycling of carbon, nitrogen, and sulfur (e.g., Kisker et al., 1999; Bittner and Mendel, 2010). Molybdenum (Mo) is an essential trace element for archea, bacteria, and eukaryotes (e.g., Williams and Fraústo da Silva, 2002; Zhang and Gladyshev, 2008; Hernandez et al., 2009). More than 60 metalloenzymes and proteins have been identified containing Mo (Lippard et al., 1994; Hille, 1996; Kisker et al., 1997; Stiefel, 1997, 1998; Kroneck and Abt, 2002; Boll et al., 2005; NC-IUB, 2012; ExPASy, 2012), including nitrogenase and nitrate reductase, which tie the element to the nitrogen cycle (e.g., Kroneck and Abt, 2002).

Although Mo is relatively scare in the Earth’s crust (1.1 ppm, Wedepohl, 1995), it is more available to biological processes than many other abundant metals in the crust (e.g., Al of 7.96%, Sr of 333 ppm, and Ti of 4,010 ppm; Wedepohl, 1995) based on water concentrations. The total dissolved Mo concentrations are relatively low in river waters (~5 nM; Martin and Meyback, 1979), whereas this trace element is the most abundant transition metal in the oxygenated ocean (dissolved Mo: 105 nM; Collier, 1985). Generally, those low-level trace elements such as Fe may potentially limit the growth of phytoplankton in the ocean, and particularly in the high nutrients and low chlorophyll *a* areas (e.g., Martin and Fitzwater, 1988; Boyd et al., 2000). Mo deficiency is not common in natural environments, and it did, however, occur for many terrestrial plants (e.g., Hewitt and Bolle-Jones, 1952; Gupta, 1997; Kaiser et al., 2005), and even for freshwater phytoplankton (Dumont, 1972; Romero et al., 2011; Glass et al., 2012). Recently, Barron et al. (2009) reported that lack of Mo may limit atmospheric N_2_ fixation in tropical forests with highly weathered acidic soils. Glass et al. (2010) further demonstrated that extremely low Mo levels (<1 nmol/L) can induce N-limitation for freshwater and coastal filamentous heterocystous cyanobacteria. Indeed, the unavailability of Mo has been long observed as limiting N_2_ fixation or nitrate assimilation in coastal waters (e.g., Brattberg, 1977). Howarth and Cole (1985) hypothesized that high levels of sulfate in seawater might competitively inhibit algal Mo uptake in coastal waters.

In enclosed basins, e.g., the Cariaco Trench, the Black Sea, and the Saanich Inlet, Mo may be depleted with concentrations of as low as 3 nmol/L, whereas the sediments there accumulated Mo as high as 140 μg/g (Berrang and Grill, 1974; Emerson and Huested, 1991). It seems that total dissolved Mo concentrations in the ancient reducing ocean might be similarly low (e.g., ~10% of the present oceanic levels, Anbar and Knoll, 2002). Furthermore, reduced Mo probably existed in the ancient reducing ocean too. This mini review summarizes the recent advances regarding the redox chemistry of Mo in natural waters. The biological involvements of reduced Mo over the Earth’s history are discussed.

## REDOX SPECIATION OF Mo IN NATURAL WATERS

The transition element Mo possesses a wide range of different redox species (+II to +VI). Under the current atmospheric *p*O_2_ of 0.2 atm, molybdate ions (e.g., ${\text{MoO}}_{4}^{2-}$ and ${\text{HMoO}}_{4}^{-}$) are the most abundant chemical forms of Mo in oxygenated freshwater and seawater systems, whilst reduced Mo(V), likely as ${\text{MoO}}_{2}^{+}$, ${\text{MoO}}_{3}^{+}$, and ${{\text{Mo}}_{2}\text{O}}_{4}^{2+}$ (e.g., Szilágyi, 1967; Loach, 1970; Bertine, 1972; Coughlan, 1980), are expected to coexist in reducing environments (Brookins, 1988). Under strongly reducing conditions, Mo is expected to be further reduced to its less soluble forms (e.g., MoO_2_ and MoS_2_). As Mo(II) and Mo(III) have never been reported in the aquatic systems (e.g., Mendel, 2005), their natural occurrences will not be discussed here.

The O atoms in ${\text{MoO}}_{4}^{2-}$ may be replaced by S in the presence of HS^-^, creating a series of thiomolybdate complexes (Helz et al., 1996). Mo in these compounds can be further reduced to Mo(IV) and Mo(V), forming a series of sulfido species (Miller et al., 1980; Vorlicek and Helz, 2002). Wang et al. (2009), using a new technique for separating Mo(V) from Mo(VI) in natural waters, quantified the Mo(V) levels in sulfidic microbial mats and even in some reducing waters (Wang et al., 2009, 2011). As a significant transient intermediate during reductive diagenesis, reduced Mo(V) can be present in specific niches, e.g., in reducing porewater. Indeed, Mo(V) might range from 5–20 nM, accounting for up to ~20% of ΣMo under low-sulfide conditions (<100 μmol/L; Wang et al., 2011). Mo(V) may be further reduced to Mo(IV), as in MoS_2_ under strongly sulfidic conditions (HS^-^ > 100 μmol/L; Wang et al., 2011).

In the present ocean, the redox switching of Mo can only occur in specific niches under bacterial mediation. For example, Mo(VI) can be reduced to the intermediate state of Mo(V), and the reduced state of Mo(IV) as molybdenite [MoS_2_(s)] by sulfate-reducing bacteria in the presence of sulfide (e.g., Tucker et al., 1997, 1998; Biswas et al., 2009). Some microorganisms can also oxidize the reduced Mo (e.g., Sugio et al., 1992), and, indeed, reduced Mo can serve as the electron donor to sustain autotrophic growth (e.g., Lyalikova and Lebedeva, 1984). In particular, Mo(V) may be produced from bio-oxidization of mineral molybdenite (MoS_2_; e.g., Brierley, 1974). Once produced, the reduced Mo(V) may be complexed and stabilized with organic ligands naturally for a long while (Szilágyi, 1967; Bertine, 1972).

The existence of reduced Mo(V) was proposed later on as a potential limiting factor for cyanobacterial productivity in coastal and oceanic surface waters (Howarth and Cole, 1985; Yamazaki and Gohda, 1990). Griffin (1975) pointed out that nitrogenase in nitrogen fixers will not be active unless a trace amount of Mo(V) complexes is present. Howarth and Cole (1985) speculated that molybdate might be reduced extracellularly, and the reduced Mo(V), instead of the total, might be responsible for cyanobacterial blooms. Indeed, specific niches like anoxic microzones widely exist in the present oxygenated ocean due to cellular exudation of reduced substances and organic colloids (e.g., Carpenter and Price, 1976; Bryceson and Fay, 1981; Paerl and Bland, 1982; Paerl, 1985; Paerl and Prufert, 1987; Ploug et al., 1997). The diazotrophic cyanobacteria *Trichodesmium* could also form anoxic microzones by aggregating together. Inside these anoxic microzomes, the reduced Mo(V) was produced and actively involved in N_2_ fixation (Howarth and Cole, 1985; Paerl, 1985; Paerl et al., 1987).

## BIOLOGICAL UPTAKE AND ASSOCIATED REDOX CHANGES OF Mo IN CELLS

In contrast to its higher abundance in the present ocean (105 nM; Collier, 1985), the biological requirement of Mo is relatively lower than many other essential elements including Fe and Cu (e.g., Finkel et al., 2006). The molar Mo/Fe ratio is only 0.03 in bacteria (Barton et al., 2007), and 0.005 in some eukaryotic phytoplankton (Ho et al., 2003). Such a relatively lower requirement of the element may be attributable to the limited numbers of Mo containing enzymes in biology (e.g., Zerkle et al., 2005; Finkel et al., 2006), though these are essential to basic biological processes (e.g., nitrogen metabolism).

**FIGURE 1:**
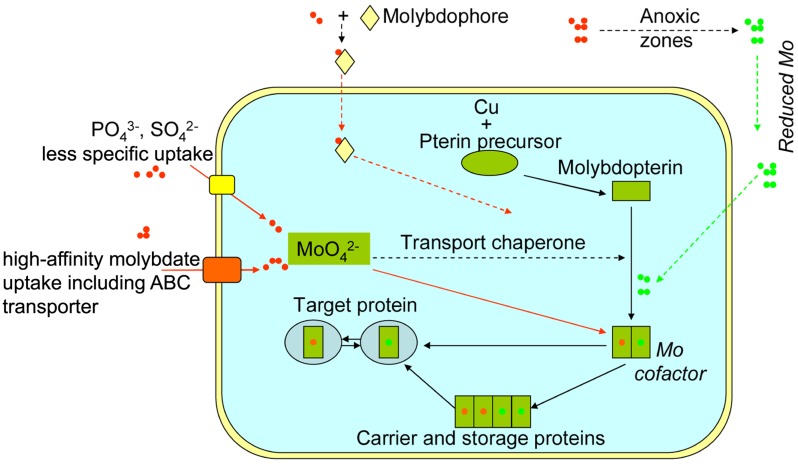
**Schematics of Mo uptake and storage in prokaryotic and eukaryotic cells.** Routes represented by dashed lines were only identified in prokaryotes, while routes represented by solid lines occurred in both current prokaryotes and eukaryotes. Green dots refer to reduced Mo species, and red dots refer to oxidized Mo (molybdate).

Until now, both less-specific and high-affinity molybdate uptake systems have been identified in biology (**Figure 1**). Previous work has confirmed that current prokaryotic and eukaryotic cells possess efficient uptake systems to utilize this element (e.g., high-affinity molybdate transporter; Tejada-Jimenez et al., 2007; Tomatsu et al., 2007; Baxter et al., 2008; Bittner and Mendel, 2010) including ABC transporter. Eukaryotic molybdate transport might involve more complex systems. In contrast, less-specific uptake of oxidized Mo widely exists in the present prokaryotic and eukaryotic cells, which utilizes other anion transporters: phosphate (Heuwinkel et al., 1992) or sulfate transporters (Tweedie and Segel, 1970; Marschner, 1995). Work has further shown an alternative: some soil bacteria are able to excrete siderophores (aminochelin) to complex extracellular Mo, and utilize trace amount of the element from ambient environments (Liermann et al., 2005).

Once inside cells, Mo cofactors are synthesized, and then allocated to the appropriate apo-enzymes via carrier proteins (Aguilar et al., 1992; **Figure 1**). These cofactors can be chaperoned to target proteins, into which they are inserted by specific trafficking proteins in prokaryotes (e.g., Ba et al., 2009; Mendel and Schwarz, 2011). Pau and Lawson (2002) reported that some bacteria possess specific molybdate-binding protein with a capacity of storing up to eight molybdate oxyanions for later use by the cells. As Mo in enzymes is extremely sensitive to intracellular oxidations such as reactive oxo species (Rajagopalan and Johnson, 1992), it is well protected within the storage proteins (e.g., Massey et al., 1970; Aguilar et al., 1992; Ichimori et al., 1999; Fenske et al., 2005; Schemberg et al., 2008; Hernandez et al., 2009; **Figure 1**). With the protection, Mo can easily switch redox states, and be actively involved in transferring electron/proton and even oxygen (e.g., Swedo and Enemark, 1979).

The Mo enzymes generally include two types of cofactors on the basis of the structure: Mo-co and Fe-Mo-co. Fe-Mo-co is a unique poly-metallic compound (MoFe_7_S_6_), which has been found only in Mo nitrogenase (e.g., Howard and Rees, 1996; Einsle et al., 2002). Two alternative nitrogenases (Fe and V) will not be discussed here. Mo nitrogenase catalyzes the ATP-dependent reduction of atmospheric dinitrogen to bioavailable ammonia, which represents the key point of entry of reduced nitrogen into the food chain (Burris, 1991; Burgess and Lowe, 1996; Hu et al., 2008). In the catalytic reaction, the N≡N triple bond is broken and therefore N_2_ is being reduced at a sterically protected, single Mo center (Fe-Mo-co; Yandulov and Schrock, 2003). Mo-co is a mononuclear Mo atom coordinated to the sulfur atoms of a pterin. The task of the pterin is to position the catalytic Mo atom correctly within the active center, to control its redox behavior, and to participate in the electron transfer to and from the Mo atom (Mendel and Bittner, 2006). Mo-co containing enzymes are ubiquitous in archae, bacteria, and eukaryotes (Williams and Fraústo da Silva, 2002; Zhang and Gladyshev, 2008; Hernandez et al., 2009), including four families: xanthine oxidase, aldehyde oxidoreductase, sulfite oxidase, and dimethylsulfoxide reductase. This mini review will only discuss a few Mo-co-containing enzymes critical in the cycling of sulfur and nitrogen. Among them, sulfite oxidase catalyzes the conversion of sulfite to sulfate, which is the terminal step in the metabolism of sulfur-containing compounds (e.g., cysteine and methionine) in bacteria, plants, and mammals (Cramer et al., 1979). Polysulfide reductase, another group of Mo-containing enzymes, converts polysulfide (as sulfur) to H_2_S (Stiefel, 1993). Nitrate reductase catalyzes the first step of nitrate reduction during nitrate assimilation for all autotrophs including higher plants and algae (e.g., Eppley et al., 1969; Butler et al., 1999; Campbell, 1999; Morozkina and Zvyagilskaya, 2007).

Mo may exist in several different redox states in these enzymes: e.g., oxidized Mo(VI), intermediate Mo(V), and reduced Mo(IV) forms. Two electron transfer or one oxygen transfer reactions are coupled with Mo(IV) oxidation to Mo(VI), and the active Mo(IV) state is regenerated by two subsequent one-electron transfer reactions through the intermediate Mo(V) state (Kisker et al., 1997). Those one-electron transfer reactions are carried out by switching the redox pairs: Mo(IV)/(V) or Mo(V)/(VI). The intermediate Mo(V) can act as an interface between one- and two-electron redox reactions, and catalyzes a variety of reactions using water or H_2_S as the electron donor (Hille, 1999, 2002). Mo(V) is generally produced from Mo(IV) by transferring a reducing equivalent or from Mo(VI) by accepting an electron (e.g., Barber et al., 1987). Hence, Mo(V) levels in cells could account for as high as 50% of the total intracellular Mo (Hille and Massey, 1985).

## INVOLVEMENT OF REDOX SPECIATION OF Mo IN THE EVOLUTION OF LIFE

**FIGURE 2:**
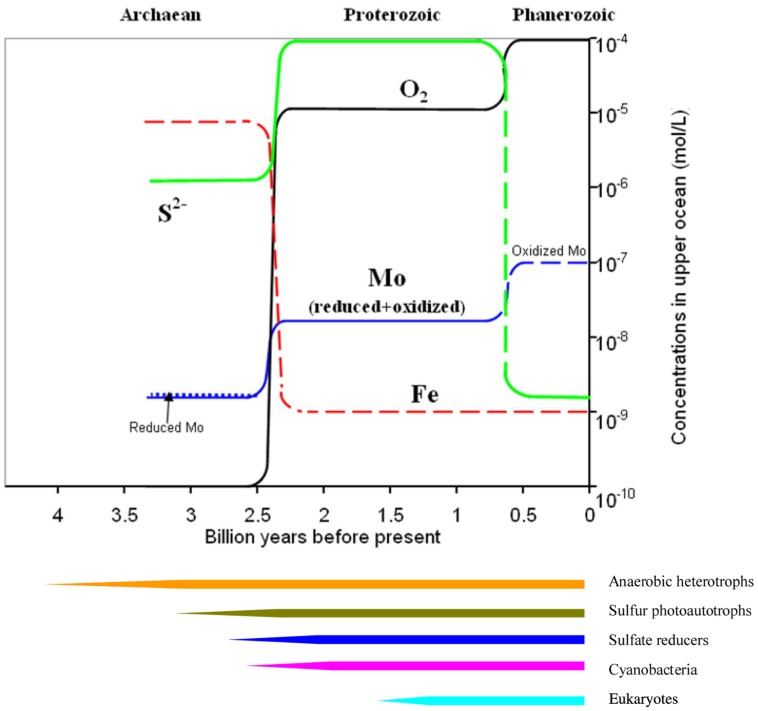
**Evolution of the Mo redox dynamics along with key elements of O, S, and Fe in the ocean over the Earth’s history.** Evolutionary sequence of major life forms is shown at the lower panel. Note: the detailed and complex evolution of both chemical composition and life forms are smoothed out for simplicity.

In the present oxygenated ocean, reduced Mo species have only been confined to specific niches including cytoplasm (e.g., Hille and Massey, 1985), sulfidic mats, and some reducing waters (Wang et al., 2009, 2011). These reduced Mo species might, however, be abundant in the ancient reducing ocean, e.g., in the ferruginous Archaean and the sulfidic Proterozoic (**Figure 2**). The existences of diverse Mo redox species probably facilitated the emergences of Mo enzymes (or prototypes) catalyzing metabolic reactions in the cycling of carbon, nitrogen, and sulfur, and finally the evolution of bacteria and eukaryotes, which possess Mo enzymes (**Figure 2**).

The Archaean ocean (3.5 ~ 2.2 billions years ago) was generally characterized by reduced species including ${\text{NH}}_{4}^{+}$, Fe(II), and a small amount of HS^-^ (Zerkle et al., 2005; Fani and Fondi, 2009). At this stage, Mo was mostly released from volcanoes and/or hydrothermal vents (Nisbet, 2000). Different redox species (IV, V, and VI) probably coexisted together under such reducing conditions. MoS_2_ has an extremely low solubility in aqueous solutions (Ksp = 10^-^^43^, Garrels and Christ, 1965), and ${\text{MoS}}_{4}^{2-}$ is easily adsorbed onto mineral particles and organic materials (Helz et al., 1996). Both processes resulted in low levels of the total dissolved Mo, and instead increased the proportion of Mo(V). The existence of intermediate Mo(V), and redox switching of Mo, therefore, faciliated the elctron transfer at this stage, and were essential in functioning of Mo-co containing enzymes, catalyzing certain reactions for carbon, nitrogen, and sulfur cycling since reduced Mo(V) are essential in N_2_ fixation (Griffin, 1975; Howarth and Cole, 1985; Yamazaki and Gohda, 1990). Yandulov and Schrock (2003) reported that all reduced species of Mo (+II to +V) are involved in catalytic reactions of N_2_ fixation in nitrogenase. As all Mo redox species (+II to +VI) probably existed in the Archaean ocean, the abundance of CO_2_ and N_2_, and lack of ${\text{NH}}_{4}^{+}$ finally prompted the emergence of Mo nitrogenase, which efficiently fixes atmospheric N_2_ to bioavailable ${\text{NH}}_{4}^{+}$. In the late Archaean ocean, as the source of electrons for the photosynthesis switched from HS^-^ to H_2_O for increased energy production, photosynthetically produced O_2_ increased accordingly (e.g., Anbar and Knoll, 2002; **Figure 2**) until a slight oxygenation of the atmosphere occurred gradually between 2.4 and 2.2 billions years ago (e.g., Farquhar et al., 2000; Kasting and Siefert, 2001; **Figure 2**).

In the Proterozoic ocean (2.2 ~ 0.6 billions years ago), terrestrial input of ${\text{SO}}_{4}^{2-}$ along with ${\text{MoO}}_{4}^{2-}$ predominated due to the slightly increased atmospheric *p*O_2_ (e.g., Anbar and Knoll, 2002). Paradoxically, sulfate-reducing bacteria also developed, and a sulfidic Proterozoic ocean was, therefore, formed at least near the shelves (e.g., Saito et al., 2003). The redox reactions of Mo between Mo(VI) and Mo(IV) at this stage were likely mediated by sulfur photoautotrophs and sulfate reducers (Anderson and Spencer, 1949). With the further increase of atmospheric *p*O_2_ in the late Proterozoic, reduced species of Mo were only confined to limited niches including sulfidic waters/sediments and microzones, whilst all oxyanions including nitrate and sulfate became abundant in the ocean. New Mo uptake and storage systems evolved in order to efficiently utilize the ambient molybdate via either high-affinity uptake or less-specific uptake. A series of Mo-co-containing enzymes were newly formed (e.g., Zerkle et al., 2005) to utilize the abundant sulfate and nitrate (e.g., Nicholas et al., 1963; Scott et al., 2008; Wille et al., 2008). New eukaryotes with more efficient molybdate uptake systems (Thiel et al., 2002; Zahalak et al., 2004) and specific storage proteins protecting the sensitive reduced Mo within cytoplasm eventually evolved along with eukaryotes on Earth probably about 1.5–1.0 billion years ago (e.g., Ba et al., 2009).

## SUMMARY

Mo has been considered as one of the most important elements dictating the evolution of life. This mini-review summarized the current findings regarding redox speciation of Mo in natural waters. The contemporary observations of reduced Mo led to the hypothesis that these reduced Mo also existed in the ancient reducing ocean (e.g., in the ferruginous Archaean and sulfidic Proterozoic). The versatile redox chemistry of Mo ranging from +II to +VI facilitates electron transfer and even oxygen transfer in reactions of carbon, nitrogen, and sulfur in biology. Similarly, redox switching of Mo might be essential in the evolution of Mo enzymes catalyzing different electron and oxygen transfer reactions.

In the ferruginous Archaean, reduced Mo such as Mo(V) might fundamentally contribute to the metabolic reactions of nitrogen and sulfur by forming nitrogenases and other Mo-containing enzymes. In the sulfidic Proterozoic, redox switching of Mo probably coupled with the sulfur cycling initially. The further increasing of photosynthetically produced O_2_ constrained reduced Mo only within specific niches including microzones, cytoplasm, and reducing sediments/waters. New eukaryotes with active uptake and storage systems developed in order to utilize oxidized molybdate, and a series of Mo-containing enzymes for nitrate assimilation and sulfur detoxification also evolved later on.

## Conflict of Interest Statement

The author declares that the research was conducted in the absence of any commercial or financial relationships that could be construed as a potential conflict of interest.
